# Prognostic and immunotherapeutic implications of histamine-related biomarkers in lung adenocarcinoma

**DOI:** 10.7717/peerj.21760

**Published:** 2026-09-30

**Authors:** Fancai Chen, Jinfeng Wang, Jicheng Yang, Yuexi Yuan, Hua Luo, Jianming Peng, Chengyi Yan, Zhang Zhang

**Affiliations:** Department of Thoracic Surgery & Cardiovascular Surgery, The Affiliated Changsha Central Hospital of University of South China, Changsha, Hunan, China

**Keywords:** Lung adenocarcinoma, Histamine, Prognostic model, Immunotherapy

## Abstract

**Background:**

Recent researches have revealed that histamine plays a critical role in promoting pathogenesis of cancers. However, the key histamine-related molecular signatures and their functional roles in lung adenocarcinoma (LUAD) remain largely unknown.

**Methods:**

Differentially expressed analysis was conducted on transcriptome data to screen differentially expressed genes (DEGs) between LUAD and control (adjacent non-cancerous samples) groups. The differentially expressed histamine-related genes in LUAD were identified by intersecting DEGs and histamine-related genes. Univariate Cox regression and least absolute shrinkage and selection operator (LASSO) analyses were applied to determine key signatures associated with LUAD prognosis. Subsequently, we constructed a histamine-related prognostic risk model, compared functional enrichment and immune cell infiltration, and predicted immunotherapeutic responses and chemotherapeutic sensitivities between two risk groups using gene set enrichment analysis (GSEA), CIBERSORT, and TIDE algorithms. Finally, we investigated the expression patterns and methylation levels of key signatures.

**Results:**

Firstly, 24 candidate histamine-related genes in LUAD, which were involved in histamine transport/secretion and neurotransmitter receptor activity were identified. After univariate Cox regression and LASSO analyses, five histamine-related genes, including SLC18A2, CDT1, PLA2G3, TSPAN7 and BTK were identified as associated with LUAD prognosis. Specifically, CDT1 was up-regulated, while the other four genes were down-regulated in LUAD samples. By calculating the histamine-related risk score, a risk model with area under curve values >0.6 (up to 0.7) for 1-, 3-, and 5-year predictions in both training and validation sets was constructed and validated. Further enrichment analysis showed a relation between risk score and immune (immune cells, immunotherapy response), and we found that low-risk group was in a more immunoactive status. Importantly, low-risk patients were predicted to have better responses to immunotherapy, while high-risk group had greater sensitivity to crizotinib, docetaxel, erlotinib, gefitinib, paclitaxel and vinorelbine. Moreover, we identified that the DNA methylation profiles of these signatures were altered in LUAD.

**Conclusion:**

In this study, we focused on histamine, identified histamine-related genes and developed a new prognostic model for LUAD, providing an important theoretical foundation and practical reference for pathogenesis and precision treatment of patients with LUAD.

## Introduction

Lung cancer remains the leading cause of cancer-related deaths worldwide. As the most common subtype of non-small cell lung cancer (NSCLC), lung adenocarcinoma (LUAD) accounts for more than 40% of cases and has the highest mortality among all malignant tumors (Sung et al., 2021). In East Asia, the proportion of LUAD is higher, accounting for about 60% of all lung cancer cases. Among non-smoking women, this subtype is the predominant pathological type (Blechter et al., 2025). Currently known risk factors comprise smoking, long-term exposure to environmental carcinogens, genetic susceptibility and chronic lung diseases (Huang et al., 2021). Early-stage LUAD typically presents without overt clinical symptoms; however, it is prone to hemorrhagic metastasis, resulting in a poor prognosis (Wu & Wang, 2019). Surgical resection remains the most effective radical treatment modality when clinical conditions permit. Although advances in screening and treatment have improved lung cancer care, late diagnosis still accounts for more than 50% of cases, which significantly affects treatment choices and outcomes (Tun et al., 2025). For this reason, the exploration of novel biomarkers and development of prognostic models is crucial for risk stratification and prognostic assessment of patients, which can contribute to precision medicine.

Histamine is a bioactive monoamine substance generated by histidine decarboxylase in various cells (*e.g.*, basophils and mast cells). It plays a significant role in embryonic development, cell proliferation and tumor growth (Moriguchi & Takai, 2020; Blaya et al., 2010). Histamine mediates a wide range of pathological physiological effects through four specific G protein-coupled receptors—histamine H1 receptor (H1R), H2 receptor (H2R), H3 receptor (H3R), H4 receptor (H4R) (Tiligada & Ennis, 2020). Specifically, H1R is widely expressed in various tissues, such as bronchial and vascular smooth muscle cells. Once activated, this receptor leads to airway contraction, vasodilation, increased vascular permeability, and enhanced mucosal secretion (Hatipoglu et al., 2023; Panettieri et al., 1990). Conversely, H2R activation is known to suppress intestinal inflammation and associated tumorigenesis, underscoring its immunomodulatory role (Branco et al., 2018; Shi et al., 2019). H3R mainly functions as a presynaptic autoreceptor in the central nervous system, inhibiting the release of histamine and other neurotransmitters, auch as glutamate, gamma-aminobutyric acid, dopamine, norepinephrine, and acetylcholine (Haas, Sergeeva & Selbach, 2008; Tiligada & Ennis, 2020). Meanwhile, H4R is mainly expressed in immune cells including eosinophils, mast cells, monocytes, dendritic cells and T cells, and it drives allergic and inflammatory responses by promoting chemotaxis (Walter, Kottke & Stark, 2011). HRs are promising targets for cancer treatment, high levels of histamine and HRs are found in many different types of tumor cells and cells in the tumor microenvironment (Nguyen & Cho, 2021). Histamine-related genes (HRGs) are involved in the formation of an immunosuppressive microenvironment and affect the immunotherapy response of hepatocellular carcinoma (Zhang et al., 2024); In LUAD, the H1R antagonist loratadine has been identified as a PP2A activator. Combining loratadine with inhibitors of STAT3, JNK, and p38 signaling pathways represents an effective strategy for inhibiting tumor growth (Chien et al., 2025). Nonetheless, the characteristics, prognostic value and potential regulatory mechanisms of HRGs in LUAD have not been systematically elucidated. As a consequence, in-depth exploration of the expression profile of HRGs in LUAD can not only provide new insights into the mechanism of tumor occurrence in LUAD, but also provide theoretical basis for building a prognosis model and developing targeted therapy.

Based on public database resources, this study integrates TCGA-LUAD dataset and GSE31210 dataset, and analyzes it with 82 HRGs. A machine learning algorithm was used to screen HRGs related to key prognosis to construct histamine-related prognosis model, and then the differences of tumor immune microenvironment, immunotherapy response and drug sensitivity between two risk patients were analyzed and compared. Moerover, the DNA methylation regulation mechanism of these key HRGs was deeply analyzed through SMART database. Finally, the quantitative real-time PCR (qPCR) technology was used to verify the clinical tissue samples of patients with LUAD and the control group, and to confirm the expression level of the selected key genes.

## Materials & Methods

### Data source

RNA sequencing data and clinical characteristics were obtained from TCGA using UCSC Xena database. Based on the clinical characteristics, LUAD samples which lacked survival information, were excluded. Ultimately, 517 samples of LUAD, along with 59 adjacent non-cancerous samples from whole-Transcriptome RNA-Seq, Illumina platform (TCGA) were used as the training cohort in the current study. The GSE31210 dataset (Affymetrix Human Genome U133 Plus 2.0 Array, 226 LUAD) was used as the validation set to validate the risk score model. We searched the MSigDB database (https://www.gsea-msigdb.org/gsea/msigdb/index.jsp) using the keyword “Histamine” and downloaded 10 Histamine-related gene sets: POS HISTAMINE RESPONSE NETWORK, POS RESPONSE TO HISTAMINE DN, POS RESPONSE TO HISTAMINE UP, GOBP CELLULAR RESPONSE TO HISTAMINE, GOBP HISTAMINE METABOLIC PROCESS, GOBP HISTAMINE SECRETION, GOBP HISTAMINE TRANSPORT, GOBP REGULATION OF HISTAMINE SECRETION BY MAST CELL, GOBP RESPONSE TO HISTAMINE, and GOMF HISTAMINE RECEPTOR ACTIVITY. After removing duplicate genes across these 10 gene sets, we obtained a total of 82 unique genes as the Histamine-related gene.

### Identification of genes related to histamine in LUAD

Firstly, based on the criteria of adjusted *p*-value < 0.05 and |log2FC| > 1, the differentially expressed genes (DEGs) between LUAD and the control groups were selected. Subsequently, the DEGs related to histamine (DE-HRGs) in LUAD were obtained by overlapping 82 HRGs and DEGs, followed by Gene Ontology (GO) and Kyoto Encyclopedia of Genes and Genomes (KEGG) enrichment analyses for DE-HRGs. Furthermore, univariate Cox regression analysis was used to screen out the genes that significantly affected the prognosis of LUAD patients from DE-HRGs according to the criteria of HR ≠ 1 and *p*-value < 0.05 with overall survival (OS) as the outcome. Then the Least Absolute Shrinkage and Selection Operator (LASSO) algorithm was used to identify the key genes of LUAD among the prognostic genes. In addition, abnormal DNA methylation as one of cancer markers will affect the gene expression profile of cancer patients (Geissler et al., 2024), affects the gene expression profiles of cancer patients. Therefore, the DNA methylation of key HRGs was comprehensively analyzed through the database of Shiny Methylation Analysis Resource Tool (SMART) database (http://smart.embl.de/). The SMART database integrates and preprocesses the DNA methylation data for TCGA-LUAD to provide standardized *β* values and probe annotation results. We used R software to further analyze the methylation *β* values obtained from the SMART database. The Wilcoxon rank-sum test was used to compare DNA methylation levels between the high-risk and low-risk groups, and the results were visualized.

### Histamine-related prognostic model

Based on the expression level of key genes in Section 2.2 and the regression coefficient of LASSO, a risk model was constructed. TCGA-LUAD patients were divided into high-risk group and low-risk group according to the median risk score. We drew Kaplan–Meier (KM) survival curves to predict survival probability between high- and low-risk groups, and we also drew receiver operating characteristic (ROC) curves for 1-, 3-, and 5-year survival to evaluate the performance of the risk score in predicting patients’ survival status. Additionally, a comprehensive clinical nomogram was built to predict the prognosis of LUAD patients. Specifically, the risk score and clinical information (age, sex, T stage, N stage and M stage) were input into univariate and multivariate Cox analysis to identify independent prognostic factors. Then, clinical nomogram was constructed by R software package “rms” to predict the one year, three years and five years survival status of patients with LUAD. The calibration curve and ROC curve were used to evaluate the performance of the prognostic nomogram.

### Analysis of immune microenvironment and therapeutic response

Firstly, differential expression analysis was conducted between the two risk groups in TCGA-LUAD patients using DEseq2. Gene Set Enrichment Analysis (GSEA) was then performed to investigate enrichment pathways of key genes, with the c2.cp.kegg.v7.5.1.symbols.gmt as reference gene set. Subsequently, the infiltration of 22 immune cell types, including B cell memory/naive/plasma, eosinophil, macrophage M0/M1/M2, mast cell activated/resting, monocyte, myeloid dendritic cell activated/resting, neutrophil, NK cell activated/resting, T cell CD4+ memory activated/resting, T cell CD4+ naive, T cell CD8+, T cell follicular helper, T cell gamma delta and T cell regulatory, was assessed using the CIBERSORT algorithm (v1.04) (Newman et al., 2015; Chen et al., 2018). Meanwhile, the enrichment scores of seven cancer-immunity cycles were calculated and compared between two risk groups. Additionally, Tumor immune dysfunction and exclusion (TIDE) score was employed to evaluate immunotherapeutic response of LUAD patients. Also, the half-maximum inhibitory concentration (IC50) values of crizotinib, gefitinib, docetaxel, erlotinib, paclitaxel and vinorelbine were calculated by “oncoPredict” R package to evaluate the sensitivity of patients in two risk groups to these six drugs.

### RNA preparation and qPCR analysis

Researchers collected LUAD tumor tissues and adjacent non-cancerous tissues from eight inpatients in the thoracic surgery department of Changsha Central Hospital, Hunan Province. Written informed consent was obtained from all participants. This study was approved the Ethics Committee of Changsha Central Hospital (Approval no. 2025-097).

The TRIzol^®^ RNA Isolation Kit (Solarbio, Beijing, China) was applied to extract RNA from tumor and adjacent control tissues. The cDNA Synthesis SuperMix for qPCR (Yeasen, Shanghai, China) was used to synthesize cDNAs to explore genes’ mRNA expression detection according to the manufacturer’s instructions. The reverse-transcribed reaction conditions included 42 °C for 2 min, 25 °C for 5 min, 55 °C for 15 min, 85 °C for 5 s and stored at 4 °C. cDNA was stored at −20 °C and used for further experiments in a week. The RT-qPCR was conducted using a qPCR SYBR Green Master Mix (Yeasen, Shanghai, China) according to construction sequence. The PCR conditions included a denaturing step at 95 °C for 3 min and 40 cycles of 95 °C for 10 s and 60 °C for 30 s for real time plate read. The relative expression of genes was performed using a 2^−ΔΔ*ct*^ method using GAPDH as the reference gene. Samples were prepared and analyzed in triplicate, and the results are presented as the mean ± SD for comparative analysis. Primer sequences used in the study are listed in Table 1.

**Table 1:** The RT-qPCR primers of key HRGs.

Primer	Sequence (5′–3′)
GAPDH-F	GATTCCACCCATGGCAAATTC
GAPDH-R	CTGGAAGATGGTGATGGGATT
SLC18A2-F	CCATGGGGGTCTTAGTGGG
SLC18A2-R	CATCCAAGAGTACCAGGGCG
CDT1-F	CTGTCGGTGGACGAGGTTTC
CDT1-R	GACGCAAGCTCAGAGATGGT
PLA2G3-F	TGTGCTCATGAGACTGCCTG
PLA2G3-R	TGGAAGACCCCCAGCTCC
TSPAN7-F	TGGATGCTGAAACTGTATGCCA
TSPAN7-R	GTCTGCATAGCGTCCGTGTA
BTK-F	AGCACAACTCTGCAGGACTC
BTK-R	CTTGATGGCCACGTCGTACT

### Statistical analysis

All statistical results were analyzed through R (version 4.4.0). The Kaplan–Meier curves and log-rank test were used to visualize and test survival difference among different groups. The differences in continuous variables between the two groups were examined by the Wilcoxon test, after Benjamini–Hochberg (BH) correction, false discovery rate (FDR)-adjusted *P*-values < 0.05 were deemed significant.

## Results

### Five HRGs were identified in the prognosis of LUAD

To identify HRGs in LUAD, we first performed differential expression analysis and identified 5,450 DEGs between LUAD and controls. Among them, 3,523 genes exhibited an up-regulated trend, while 1,927 genes demonstrated a down-regulated trend in LUAD samples (Fig. 1A). Next, 24 DE-HRGs between LUAD and controls were obtained by overlapping 82 histamine genes and 5,450 DEGs (Fig. 1B). The enrichment results from GO showed that these genes were enriched in biological processes such as histamine transport/secretion, signal release; cellular compound such as clathrin-sculpted vesicle, GABA-A receptor complex; molecular function such as neurotransmitter receptor activity, GABA-gated chloride ion channel activity (Fig. 1C). KEGG analysis revealed that they were participated in neuroactive ligand–receptor interaction, cAMP signaling pathway, amphetamine addiction and serotonegic synapse (Fig. 1D). These findings further demonstrated that the identified genes were related to histamine and nervous system.

**Figure 1:**
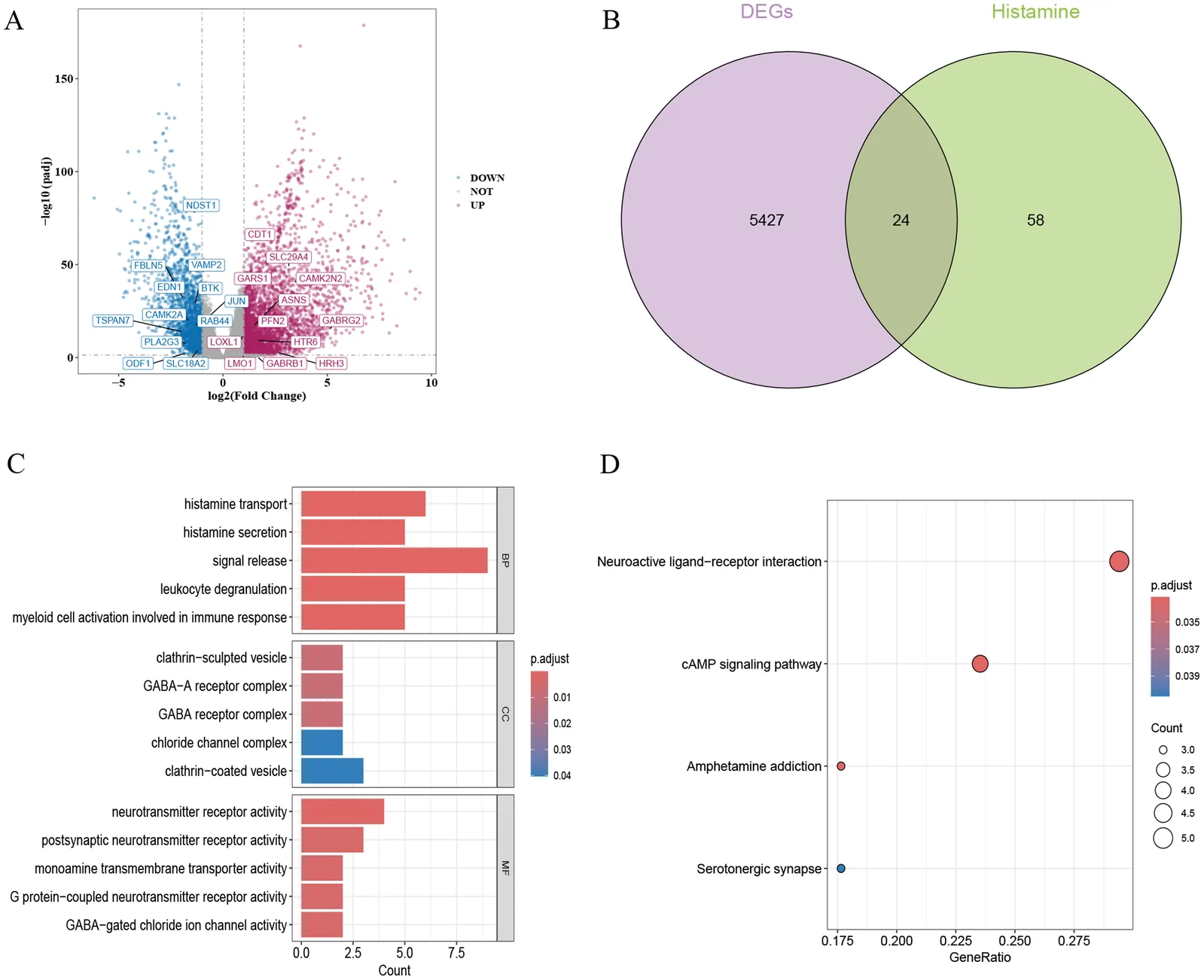
Identification of differentially expressed histamine-related genes (DE-HRGs). (A) The volcano plot of differentially expressed genes (DEGs) from the TCGA-LUAD. (B) The venn plot of DE-HRGs between DEGs and HRGs. (C) Gene ontology (GO) enrichment analysis of DE-HRGs. (D) Kyoto encyclopedia of genes and genomes (KEGG) enrichment analysis of DE-HRGs.

Next, to explore prognostic value of these genes, we first performed univariate Cox regression analysis, and found that RAB44, SLC18A2, BTK, PLA2G3, VAMP2, TSPAN7, CDT1 and GARS1 were related to LUAD survival (Fig. 2A). To further identify key prognostic signature, these eight genes were input into LASSO regression analysis. After cross-validation, SLC18A2, CDT1, PLA2G3, TSPAN7 and BTK were retained at the optimal lambda value (*λ* = 0.01252226) and used for downstream analyses (Fig. 2B). The coefficients of SLC18A2, CDT1, PLA2G3, TSPAN7 and BTK were −0.18828966, 0.11886616, −0.15872713, −0.07700973, −0.22217380, respectively (Table 2).

**Figure 2:**
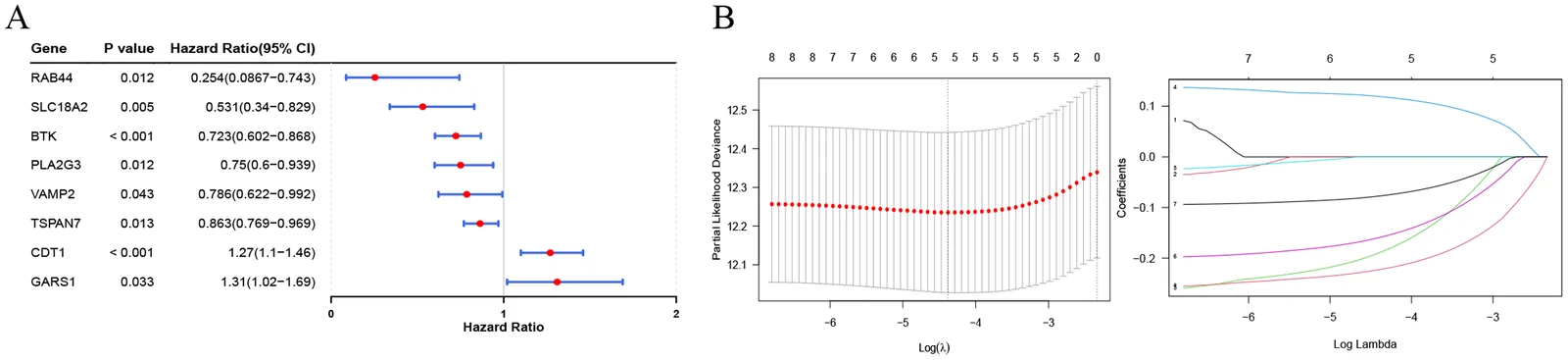
Screen of key HRGs. (A) Univariate Cox regression results indicating the relationship of DE-HRGs with survival outcomes. (B) LASSO coefficient profile plots of the eight differential prognostic HRGs.

**Table 2:** The LASSO coefficients of key HRGs.

Gene	Coef
SLC18A2	−0.18828966
CDT1	0.11886616
PLA2G3	−0.15872713
TSPAN7	−0.07700973
BTK	−0.2221738

### A histamine-related prognostic model was developed in the LUAD

According to the gene expression and coefficient, risk score of each LUAD patient was calculated. We found that high-risk (risk score > −0.49872927) patients had poor overall survival, and the area under curve (AUC) values of the risk model were 0.70 in one year, 0.65 in three years and 0.62 in five years, respectively, indicating the accuracy of the risk model (Fig. 3A). After the same analysis in validation set, the ROC curve verified the reliability of the risk score in predicting the five years survival status of LUAD patients (AUC = 0.70) (Fig. 3B). Besides, we found that significant differences in risk scores across different ages, genders, T stage, and N stage (Fig. 3C). Furthermore, to enhance the predictive power and clinical applicability of key genes, we performed uni- and multivariate Cox analyses, and found that risk score and T/N/M stage may act as independent prognostic factors in LUAD (Figs. 4A–4B). Thus, we integrated risk score with T/N/M stage to build a nomogram to predict survival outcomes of LUAD patients (Fig. 4C). Results of calibration and ROC curves (AUC = 0.74 for one year, 0.72 for three years and 0.68 for five years) indicated that the nomogram exhibited good predictive performance (Figs. 4D–4E).

**Figure 3:**
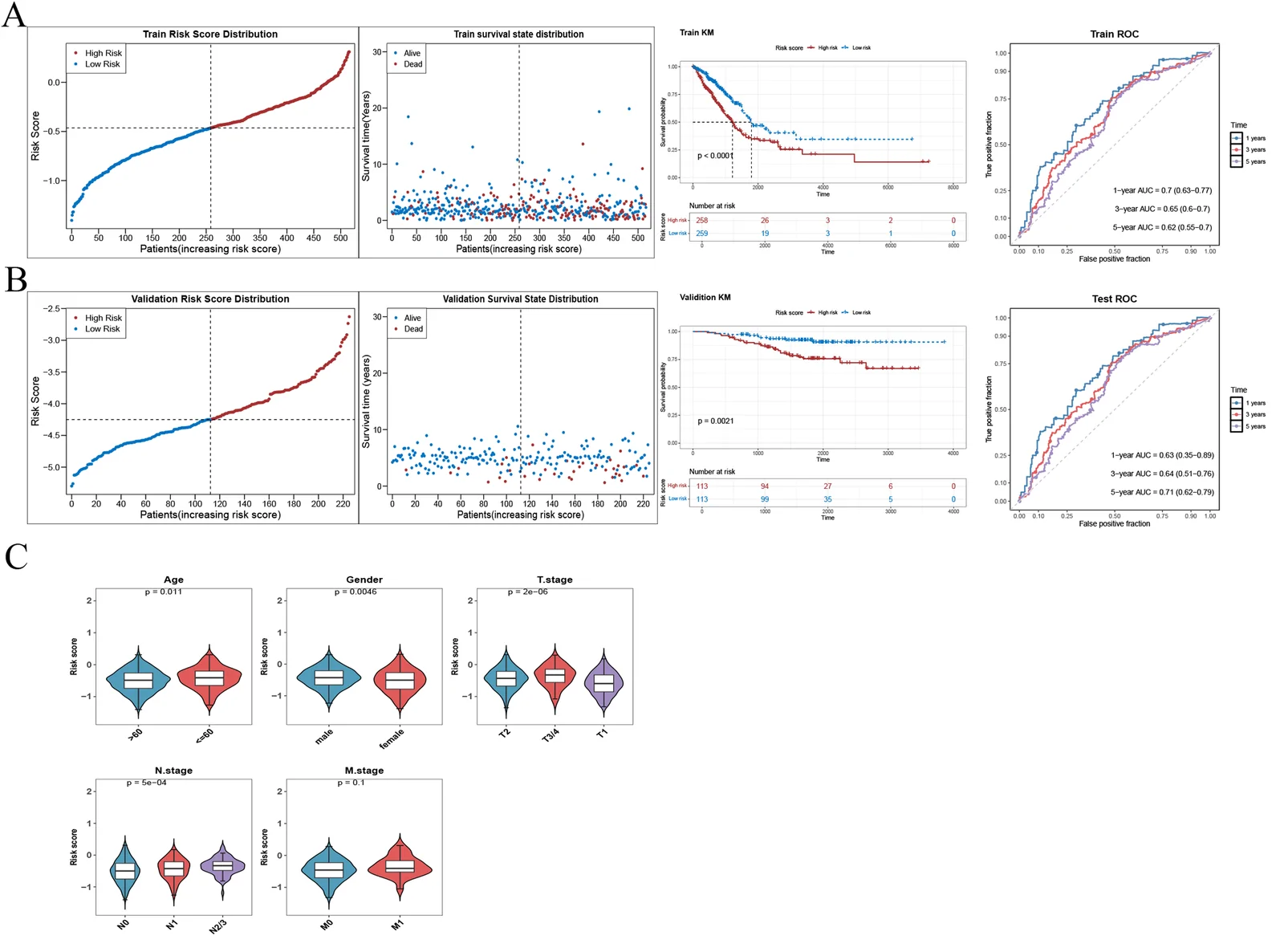
Establishment and verification of a risk model. (A) ROC curves of training set risk score plots, survival status plots, and 1-, 3-, and 5-year risk scores. (B) ROC curves for the validation set risk score plot, survival status plot, and 1-, 3-, and 5-year risk scores. (C) The relationship between clinical features and risk score.

**Figure 4:**
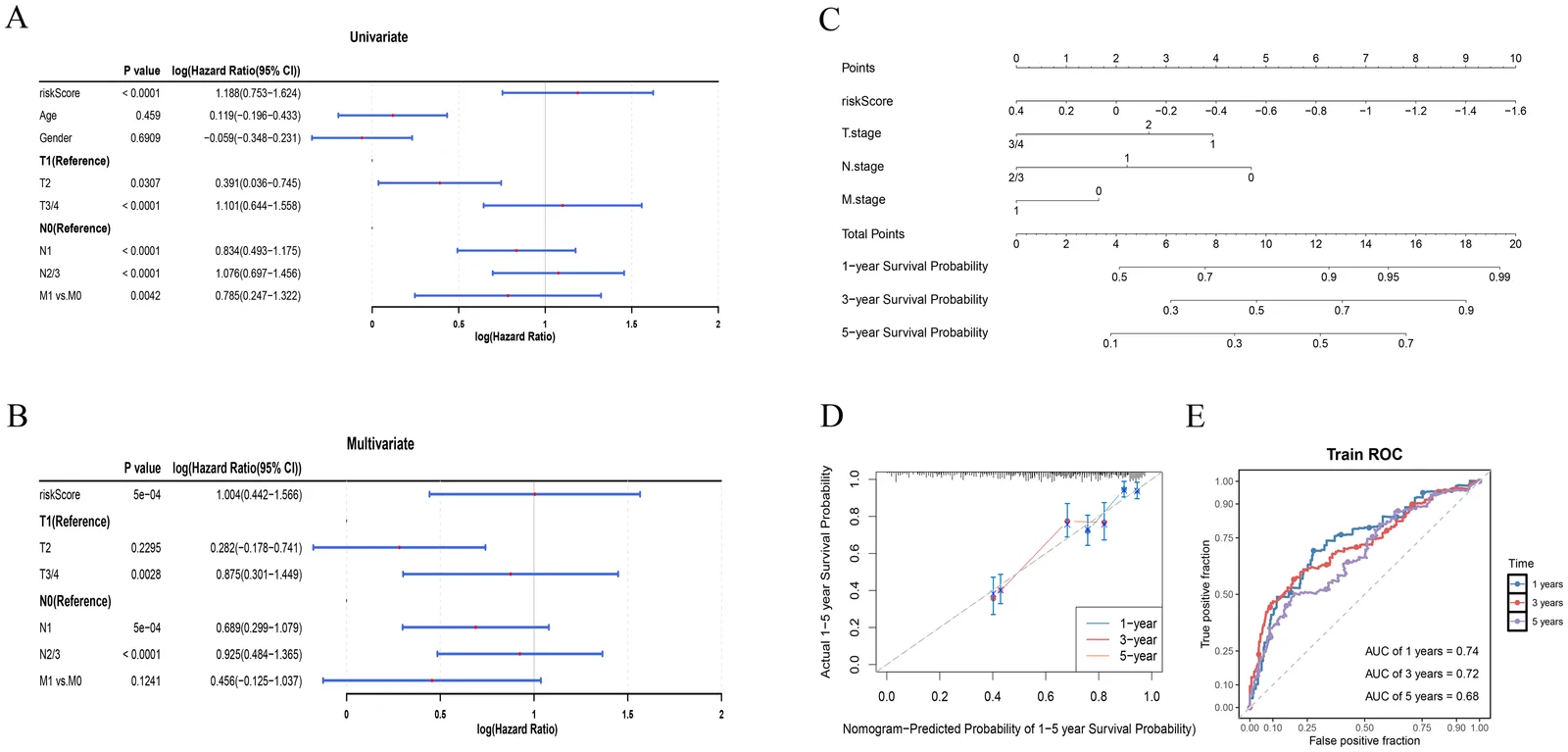
Evaluation of the independence of the prognostic model. (A) Forest plot for univariate Cox regression analysis combining riskscore with clinical indicators. (B) Forest plot for multivariate Cox regression analysis combining risk score with clinical indicators. (C) Nomogram of riskscore combined with clinicopathological factors. (D) Calibration curves for 1 year, 3 years, and 5 years correction. (E) ROC curves of nomogram at 1 year, 3 years, and 5 years.

### The two histamine-related risk groups have different tumor microenvironment and therapeutic responses

To better understand the potential mechanisms, we performed GSEA analysis in two risk groups. We found that intestinal immune network for IgA production, cell adhesion molecules, cytokine-cytokine receptor interaction and neuroactive ligand–receptor interaction were enriched in high-risk group, whereas ribosome, spliceosome, DNA replication and cell cycle were enriched in low-risk group (Fig. 5A). These results indicated that cell behaviors such as proliferation, attaching, spreading and adhesion, and immune microenvironment may differ between two risk groups. Thus, we then compared the infiltrations of immune cells between the two risk groups. It was found that the abundances of M2 macrophages, monocytes, activated mast cells, resting myeloid dendritic cells, CD4+ memory T cells and resting neutrophils were much higher in low-risk group, whereas activated CD4+ memory T cells, M0 macrophages, plasma B cells and follicular helper T cells were significantly higher in high-risk group (Figs. 5B–5C). These results demonstrated the different immune microenvironment between the two histamine groups. Different immune microenvironments may lead to different immune outcomes that the enrichment score of step-1 release of cancer cell antigens showed a significant upregulation trend, while step2–step7 exhibited significant downregulation trends in high-risk group (Fig. 5D). These findings indicated that patients in the low-risk group may be in a more immunoactive state. Thus, we then examined the immunotherapeutic response, which is affected by immune microenvironment. We found that high-risk patients had significantly higher exclusion score and TIDE score (Fig. 6A), indicating worse responses to immunotherapy. In addition, we also detected that LUAD patients in low-risk group were more sensitive to crizotinib, docetaxel, erlotinib, gefitinib, paclitaxel and vinorelbine (Fig. 6B). These results suggest that the two histamine-related subgroups exhibit divergent immune phenotypes and may require distinct therapeutic interventions to optimize clinical benefit.

**Figure 5:**
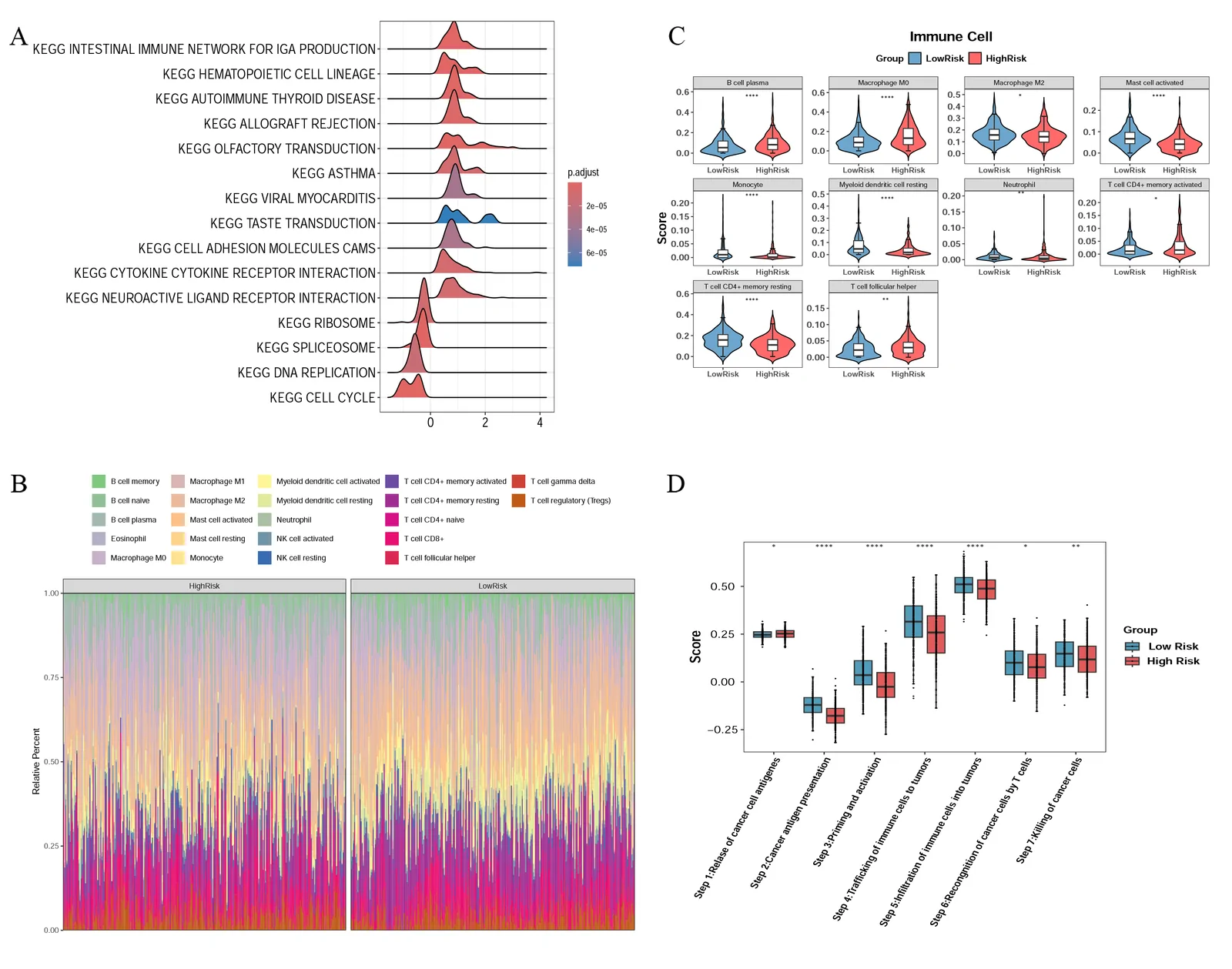
The correlation between key HRGs and immune microenvironment. (A) Gene set enrichment analysis (GSEA) of key HRGs. (B) Histogram of the proportion of 22 immune cells in LUAD samples. (C) The expression of 10 immune cells in low-risk and high-risk groups based on the TCGA dataset. (D) In TCGA, Tracking Tumor Immunophenotype (TIP) predicted the difference in tumor immune cycle status between high-risk and low-risk. *p < 0.05; **p < 0.01; ***p < 0.001; ****p < 0.0001.

**Figure 6:**
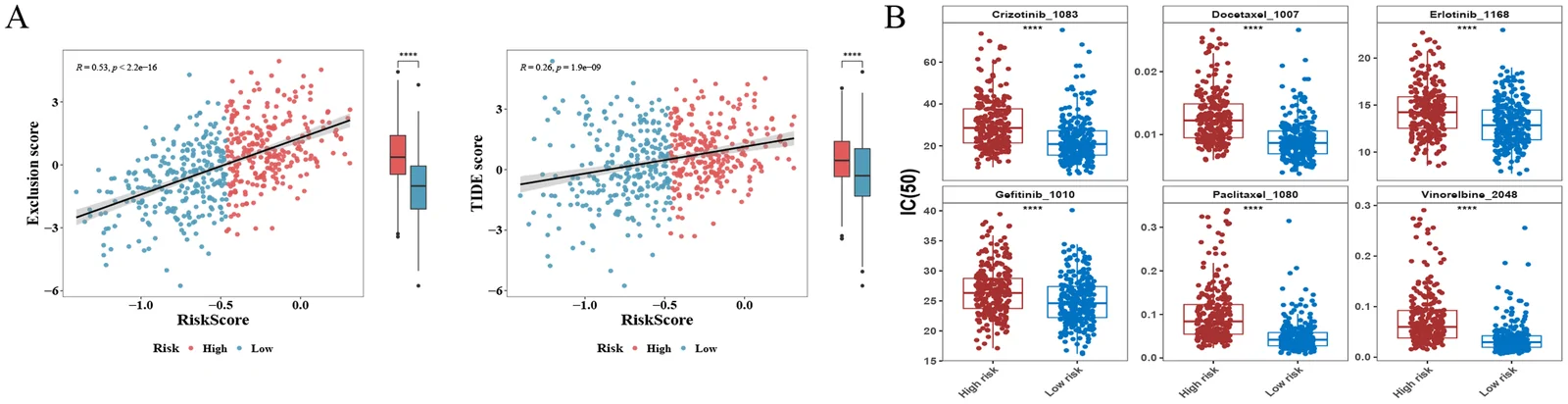
Immunotherapy and drug sensitivity prediction analysis based on gene risk score grouping. (A) The exclusion score and TIDE score in different risk score group. (B) Drug sensitivity prediction for patients in high and low risk score groups. *p < 0.05; **p < 0.01; ***p < 0.001; ****p < 0.0001.

### The expression and DNA methylation status of key histamine signatures were altered in LUAD

The expression levels of BTK, PLA2G3, SLC18A2 and TSPAN7 demonstrated downregulated trends, while CDT1 exhibited an upregulated trend in TCGA-LUAD group (Fig. 7A). The expression trends of five key genes determined by qPCR in tumor and control samples were consistent with the sequencing results from TCGA. Among them, PLA2G3 and TSPAN7 exhibited significant differences between tumor and control tissues (Fig. 7B). Additionally, we analyzed the DNA methylation of key HRG signatures. As shown in Fig. 8, SLC18A2 contained 16 CpG loci (8 in CpG island, 3 in N-shore, 1 in S-shore, 1 in S-shelf). Among these, 12 loci showed significantly upregulated trend, 2 showed significantly downregulated trend in TCGA-LUAD. The CDT1 gene contained 16 CpG loci (5 in CpG island, 5 in S-shore, 4 in S-shelf, 1 in N-shelf, and 1 in N-shore). Among them, significant upregulation was observed at cg00202760, while downregulation was evident at cg04376887, cg08255475, and cg09161138 in the TDGA-LUAD group compared to the normal group. The PLA2G3 gene harbored nine CpG loci. In the tumor group, 3 of these loci exhibited significant upregulation, and 3 showed significant downregulation when compared to the normal group. The TSPAN7 gene encompassed 12 CpG loci, with 9 situated in N-shore, 1 in S-shore, and 1 in CpG island. Notably, cg12481479 displayed significant upregulation, whereas cg20450764 and cg24088023 showed significant downregulation in the TCGA-LUAD group. The BTK gene comprised 19 CpG loci, distributed as 8 in CpG island, 2 in N-shore, 2 in N-shelf, 2 in S-shore, and 1 in S-shelf. In the TCGA-LUAD group, cg03181214 and cg21909660 demonstrated significant upregulation, while cg00126698, cg08355317, cg15847896, cg16397002, and cg27235557 exhibited significant downregulation compared to the normal group (Figs. 8A–8B). Furthermore, Spearman correlation analysis between the expression of key genes and methylation beta-values at specific loci revealed their relationship with methylation-related functions (Fig. S1). More experiments are needed to verify these DNA methylation sites and their role in regulating the expressions of key signatures. Moreover, we found that the expressions of these signatures were associated with TNM stages (Fig. 9), further supporting their important role in LUAD.

**Figure 7:**
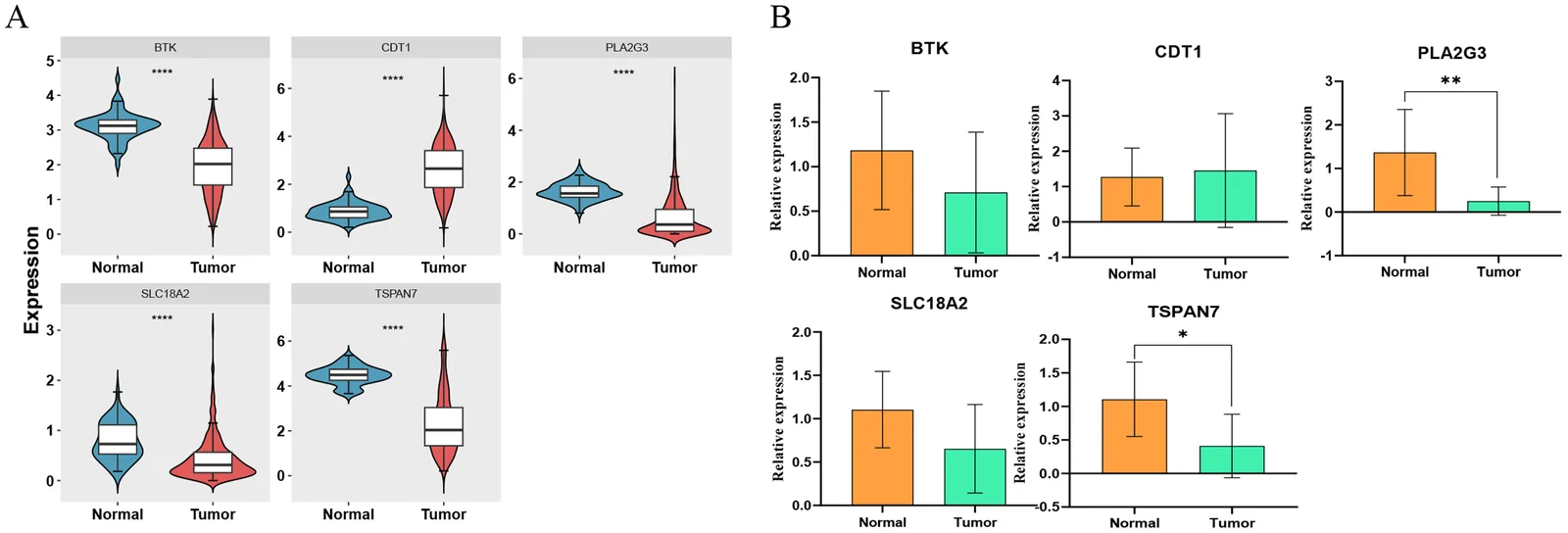
The expression level of key HRGs. (A) The expression level of key HRGs in normal and tumor groups. (B) The mRNA levels of key HRGs were determined by RT-qPCR. *p < 0.05; **p < 0.01; ***p < 0.001; ****p < 0.0001.

**Figure 8:**
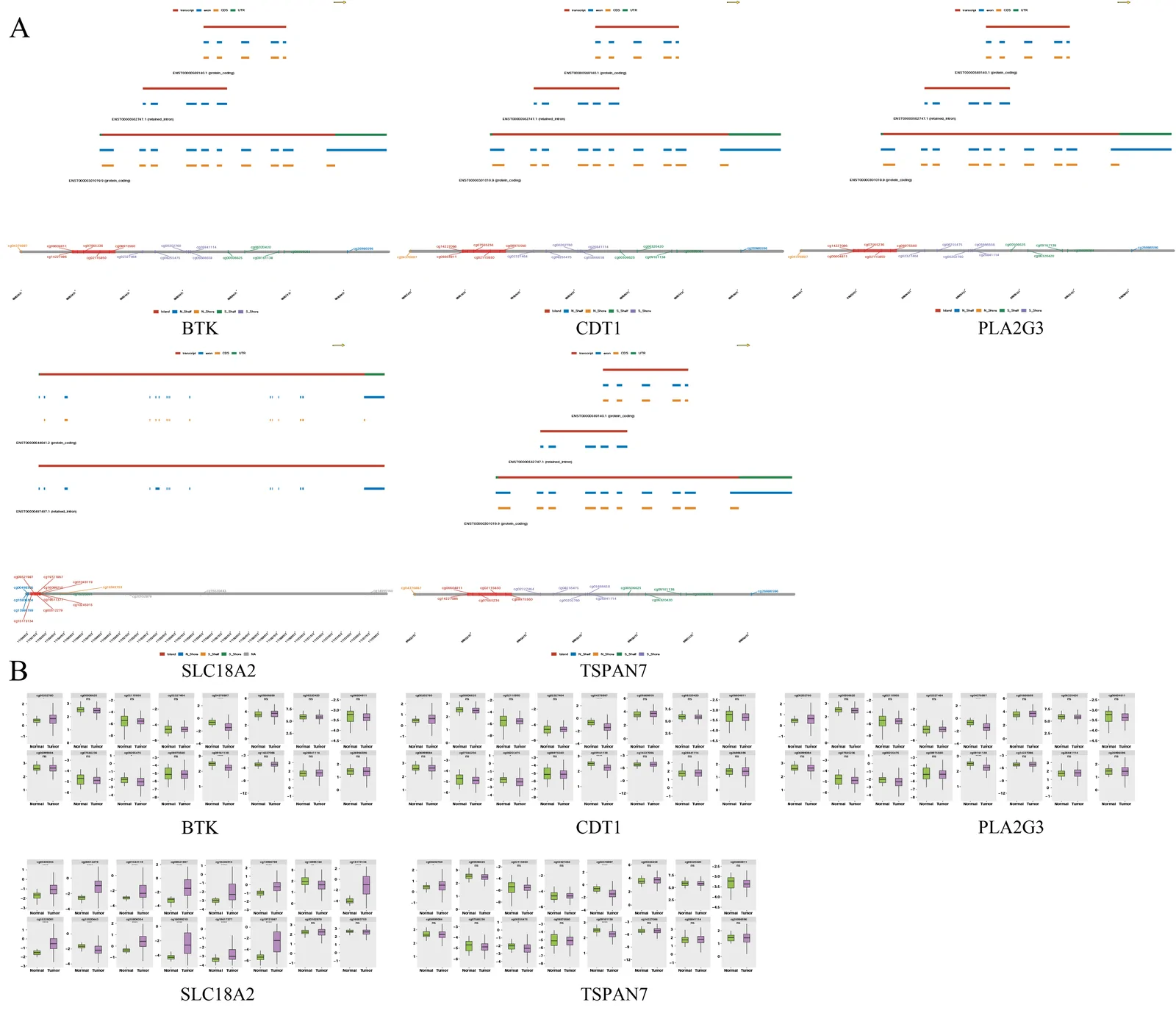
Methylation alteration of key HRGs in LUAD. (A) Detailed chromosomal distribution of the methylation probes associated with key HRGs. (B) Expression levels of methylation probes for key HRGs in the normal and tumor groups. *p < 0.05; **p < 0.01; ***p < 0.001; ****p < 0.0001.

**Figure 9:**
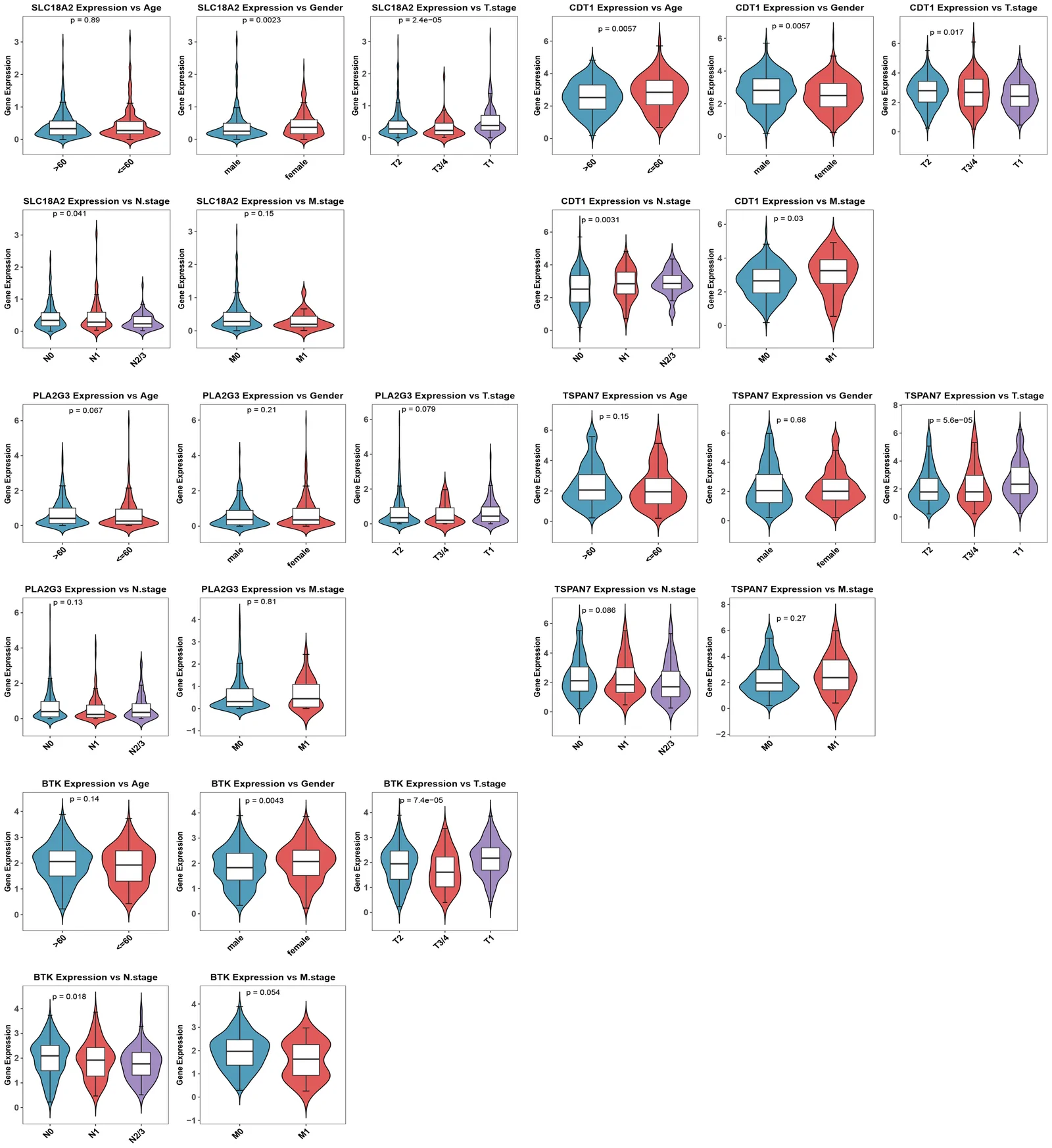
The relationship between key HRGs and clinical features (age, gender, T stage, N stage, M stage).

## Discussion

LUAD, as the most common type of lung cancer, accounts for approximately 35% to 40% of all lung cancer patients. Despite the advancements in treatment methods in recent years, the survival rate of LUAD patients remains unsatisfactory (Wei et al., 2023). Histamine, as an important mediator in the allergic inflammatory process, may be involved in regulating the repair of lung tissue injuries and the development of fibrotic lesions (Kohyama et al., 2010). Previous research data indicate that histamine is associated with the pathogenesis of lung cancer—the level of histamine in the plasma of lung cancer patients is significantly lower than that of healthy individuals (Della Rovere et al., 2006).

In our study, SLC18A2, CDT1, PLA2G3, TSPAN7 and BTK were identified as key HRGs in LUAD. The risk prognosis model demonstrates robust predictive efficacy. There are significant differences in immune microenvironment characteristics between the two risk groups, specifically manifested in immune cell infiltration (such as M2 macrophages and activated mast cells) and the seven fundamental steps of the cancer-immunity cycle. The low-risk group shows higher sensitivity to immunotherapy, and may have a stronger therapeutic response to various chemotherapy drugs (including Docetaxel, Gefitinib, Paclitaxel, Crizotinib, Erlotinib, and Vinorelbine). Finally, the qPCR analysis of clinical samples verified the expression patterns of key genes.

Specifically, BTK is a key mediator of B-cell receptor signal transduction and is crucial for the development and maturation of B cells (Da Cunha-Bang & Niemann, 2018). It has become an important target for therapeutic intervention. For instance, small molecule inhibitors such as ibrutinib have been used to treat chronic lymphocytic leukemia and specific lymphomas, which are B-cell malignancies (Tsukada et al., 1993). Besides the physiological functions of B cells, BTK is also involved in allergic and inflammatory responses. Inhibiting the activity of BTK can simultaneously block the release of histamine by anti-IgE antibodies and allergens-induced basophilic granulocytes (Smiljkovic et al., 2017). Similarly, the downregulation of BTK in mast cells significantly reduces the secretion of pro-inflammatory mediators, highlighting its direct role in allergic reactions (Heinonen, Smith & Nore, 2002). In LUAD, low-level BTK expression is associated with poor prognosis of patients. Experimental models have shown that BTK deficiency promotes tumor proliferation, invasion and metastasis (Huang et al., 2025). Moreover, the downregulation of BTK in LUAD is associated with changes in immune infiltration characteristics and changes in checkpoint expression, suggesting its involvement in regulating the tumor immune microenvironment, which may provide a reference for immunotherapy strategies (Jiao et al., 2024). Our research results are highly consistent with the above observation conclusions.

SLC18A2 (also known as vesicular monoamine transporter 2 (VMAT2)) is a presynaptic membrane protein that is responsible for encapsulating dopamine, serotonin, norepinephrine, and histamine into synaptic vesicles for subsequent release (Cliburn et al., 2017). This protein participates in the synthesis and storage of histamine within hematopoietic cells by promoting the enrichment of monoamine neurotransmitters in the vesicles (Watson et al., 1999; Dimaline & Struthers, 1996; Merickel & Edwards, 1995). In cancer models, although VMAT1 and VMAT2 are expressed in SCLC cell lines, they are completely absent in A549 and other LUAD cells (Graff et al., 2001). Moreover, functional enrichment analysis further reveals its association with neurotransmitter-related pathways, suggesting that SLC18A2 may affect immune responses and tumor cell behavior by regulating the levels of signal molecules such as histamine.

PLA2G3 is a member of the phospholipase A2 (PLA2) gene family. All PLA2s catalyze the hydrolysis of fatty acids at the sn-2 position of glycerophospholipids, yielding free fatty acids and lysophospholipids (Dennis et al., 2011). Secreted mammalian PLA2s play key roles in dietary phospholipid metabolism, skin homeostasis and host defense against bacterial and viral pathogens (Murakami & Lambeau, 2013). Defective mast cell maturation resulting from PLA2G3 deficiency is restored by supplementation with lysophosphatidic acid 1 agonists or PLA2G3-modified extracellular vesicles (Taketomi et al., 2024). In our study, the expression of PLA2G3 was significantly decreased in LUAD group. Moreover, the infiltration level of activated mast cells in the high-risk group also demonstrated a notably declining trend, which is consistent with previous research findings. Intriguingly, emerging evidence suggests that PLA2G3 exerts multifaceted functions in lung cancer, potentially relating with processes such as chronic intermittent hypoxia (Chen et al., 2024), myeloid cell differentiation (Wu et al., 2024), lysosomal activity (Song et al., 2023), programmed cell death (Yu et al., 2025). It is postulated that PLA2G3 is critically involved in LUAD prognosis, likely through modulating the immune microenvironment.

TSPAN7, a member of the tetrameric membrane protein family, has sufficient evidence indicating its involvement in the tumor development process (Huang et al., 2022; Garcia-Mayea et al., 2022). This protein exhibits context-dependent effects in the cancer pathogenesis: in renal clear cell carcinoma and glioma, TSPAN7 enhances the cell invasion ability by activating the FAK-Src-Ras-ERK1/2 pathway and induces epithelial-mesenchymal transition (EMT), thereby promoting tumor invasiveness (Gulati et al., 2014; Shao et al., 2022). Its expression level is negatively correlated with immune checkpoint molecules (PD-1, PD-L1, CTLA-4), suggesting its potential for immune regulation in glioma (Chen et al., 2023). Conversely, in NSCLC, TSPAN7 exerts tumor suppressor function by inhibiting the EMT pathway (Wang et al., 2018). Supporting this tumor-suppressive role, our qPCR analysis confirmed significant TSPAN7 downregulation in LUAD tissues, highlighting its potential as both a prognostic biomarker and a therapeutic target in this malignancy.

CDT1, a key protein of the pre-replication complex and a DNA replication factor 1, is often abnormally expressed in various human cancers. The dysregulation of the CDT1-Geminin axis can lead to DNA replication defects and genomic instability, thereby promoting tumor formation (Petropoulou et al., 2008; Petropoulos et al., 2023). In the field of lung cancer, the expression level of CDT1 is significantly higher than that in normal tissues, and it is associated with poor prognosis of LUAD (Jiang et al., 2023). Mechanistic studies have shown that its expression is positively correlated with the transcription factor E2F, and functional experiments have confirmed that knocking down CDT1 can inhibit the proliferation of LUAD cells (Lin et al., 2024). Our research further indicates that the upregulated expression of CDT1 in LUAD is significantly related to poor prognosis, and it drives tumor progression by promoting proliferation and metastasis.

## Conclusions

This study constructed a histamine-related prognostic model for LUAD using multi-dimensional methods. We found that the core genes affect the pathogenesis of LUAD by regulating immune cell infiltration, the EMT process, and the interaction of immune checkpoints. Additionally, we identified six potential therapeutic drugs, providing molecular evidence for their application in the treatment of LUAD.

## Limitations

Although the expression of key genes was verified by qPCR, the sample size limitation of public databases remains a constraint for this study. Therefore, future research needs to further validate the biological functions and regulatory mechanisms of these genes through animal experiments.

##  Supplemental Information

10.7717/peerj.21760/supp-1 Supplemental Information 1 Rscripts

10.7717/peerj.21760/supp-2 Supplemental Information 2 Raw PCR data

10.7717/peerj.21760/supp-3 Supplemental Information 3 MIQE Checklist

10.7717/peerj.21760/supp-4 Supplemental Information 4 STROBE checklist

10.7717/peerj.21760/supp-5 Figure S1 Spearman correlation between gene expression and DNA methylation beta-values at specific CpG sites

10.7717/peerj.21760/supp-6 Supplemental Information 6 Flowchart

## Additional Information and Declarations

### Data Availability

The following information was supplied regarding data availability: The raw code files are available in the Supplemental Files.
