# Supplementary material for: Prognostic and immunotherapeutic implications of histamine-related biomarkers in lung adenocarcinoma
Source: PeerJ. 2026 Sep 30;14:e21760. doi: 10.7717/peerj.21760 (PMC13633689; doi:10.7717/peerj.21760)
Supplement: Supplementary file 1 [file peerj-14-21760-s004.pdf]

STROBE Statement—checklist of items that should be included in reports of observational studies

	Item No.	Recommendation	Page No.	Relevant text from manuscript
**Title and abstract**	1	(*a*) Indicate the study's design with a commonly used term in the title or the abstract	1-2	Prognostic and immunotherapeutic implications
		(*b*) Provide in the abstract an informative and balanced summary of what was done and what was found	1-2	five histamine-related genes, including SLC18A2, CDT1, PLA2G3, TSPAN7 and BTK were identified as associated with LUAD prognosis.
**Introduction**				
Background/rationale	2	Explain the scientific background and rationale for the investigation being reported	2-3	the key histamine-related molecular signatures and their functional roles in LUAD remain largely unknown.
Objectives	3	State specific objectives, including any prespecified hypotheses	2-3	Prognostic and immunotherapeutic implications of histamine-related biomarkers
**Methods**				
Study design	4	Present key elements of study design early in the paper	3-5	TCGA database、GSE31210 dataset
Setting	5	Describe the setting, locations, and relevant dates, including periods of recruitment, exposure, follow-up, and data collection	3-5	TCGA database、GSE31210 dataset
Participants	6	(*a*) *Cohort study*—Give the eligibility criteria, and the sources and methods of selection of	3-5	TCGA database、

		participants. Describe methods of follow-up<br>*Case-control study*—Give the eligibility criteria, and the sources and methods of case ascertainment and control selection. Give the rationale for the choice of cases and controls<br>*Cross-sectional study*—Give the eligibility criteria, and the sources and methods of selection of participants		GSE31210 dataset
		(*b*) *Cohort study*—For matched studies, give matching criteria and number of exposed and unexposed<br>*Case-control study*—For matched studies, give matching criteria and the number of controls per case	3-5	TCGA database、GSE31210 dataset
Variables	7	Clearly define all outcomes, exposures, predictors, potential confounders, and effect modifiers. Give diagnostic criteria, if applicable	3-5	TCGA database、GSE31210 dataset
Data sources/ measurement	8*	For each variable of interest, give sources of data and details of methods of assessment (measurement). Describe comparability of assessment methods if there is more than one group	3-5	Not application
Bias	9	Describe any efforts to address potential sources of bias	3-5	Not application
Study size	10	Explain how the study size was arrived at	3-5	Not application
Quantitative variables	11	Explain how quantitative variables were handled in the analyses. If applicable, describe which groupings were chosen and why	3-5	Not application
Statistical methods	12	(*a*) Describe all statistical methods, including those used to control for confounding	3-5	Kaplan-Meier curves and log-rank test
		(*b*) Describe any methods used to examine subgroups and interactions	3-5	Kaplan-Meier curves and log-rank test
		(*c*) Explain how missing data were addressed	3-5	Deletation
		(*d*) *Cohort study*—If applicable, explain how loss to follow-up was addressed<br>*Case-control study*—If applicable, explain how matching of cases and controls was addressed<br>*Cross-sectional study*—If applicable, describe analytical methods taking account of sampling strategy	3-5	Not application
		(*e*) Describe any sensitivity analyses	3-5	Not application
**Results**				
Participants	13*	(a) Report numbers of individuals at each stage of study—eg numbers potentially eligible, examined for eligibility, confirmed eligible, included in the study, completing follow-up, and analysed	6-8	TCGA database、GSE31210 dataset

		(b) Give reasons for non-participation at each stage	6-8	TCGA database、GSE31210 dataset
		(c) Consider use of a flow diagram	6-8	Not application
Descriptive data	14*	(a) Give characteristics of study participants (eg demographic, clinical, social) and information on exposures and potential confounders	6-8	TCGA database、GSE31210 dataset
		(b) Indicate number of participants with missing data for each variable of interest	6-8	TCGA database、GSE31210 dataset
		(c) *Cohort study*—Summarise follow-up time (eg, average and total amount)	6-8	TCGA database、GSE31210 dataset
Outcome data	15*	*Cohort study*—Report numbers of outcome events or summary measures over time	6-8	TCGA database、GSE31210 dataset
		Case-control study—Report numbers in each exposure category, or summary measures of exposure	6-8	TCGA database、GSE31210 dataset
		Cross-sectional study—Report numbers of outcome events or summary measures	6-8	TCGA database、GSE31210 dataset
Main results	16	(*a*) Give unadjusted estimates and, if applicable, confounder-adjusted estimates and their precision (eg, 95% confidence interval). Make clear which confounders were adjusted for and why they were included	6-8	TCGA database、GSE31210 dataset
		(*b*) Report category boundaries when continuous variables were categorized	6-8	the median risk score
		(*c*) If relevant, consider translating estimates of relative risk into absolute risk for a meaningful time period	6-8	Not application
Other analyses	17	Report other analyses done—eg analyses of subgroups and interactions, and sensitivity analyses	6-8	Not application
**Discussion**				
Key results	18	Summarise key results with reference to study objectives	9-11	SLC18A2, CDT1, PLA2G3, TSPAN7 and BTK were identified as key HRGs in LUAD.
Limitations	19	Discuss limitations of the study, taking into account sources of potential bias or imprecision. Discuss both direction and magnitude of any potential bias	9-11	the sample size limitation of public databases remains a constraint for

				this study.
Interpretation	20	Give a cautious overall interpretation of results considering objectives, limitations, multiplicity of analyses, results from similar studies, and other relevant evidence	9-11	future research needs to further validate the biological functions and regulatory mechanisms of these genes through animal experiments.
Generalisability	21	Discuss the generalisability (external validity) of the study results	9-11	molecular evidence for their application in the treatment of LUAD
**Other information**				
Funding	22	Give the source of funding and the role of the funders for the present study and, if applicable, for the original study on which the present article is based	12	grant number: 20254901

*Give information separately for cases and controls in case-control studies and, if applicable, for exposed and unexposed groups in cohort and cross-sectional studies.

Note: An Explanation and Elaboration article discusses each checklist item and gives methodological background and published examples of transparent reporting. The STROBE checklist is best used in conjunction with this article (freely available on the Web sites of PLoS Medicine at http://www.plosmedicine.org/, Annals of Internal Medicine at http://www.annals.org/, and Epidemiology at http://www.epidem.com/). Information on the STROBE Initiative is available at www.strobe-statement.org.
