# Supplementary material for: Prognostic and immunotherapeutic implications of histamine-related biomarkers in lung adenocarcinoma
Source: PeerJ. 2026 Sep 30;14:e21760. doi: 10.7717/peerj.21760 (PMC13633689; doi:10.7717/peerj.21760)
Supplement: Supplementary file 2 [file peerj-14-21760-s005.pdf]

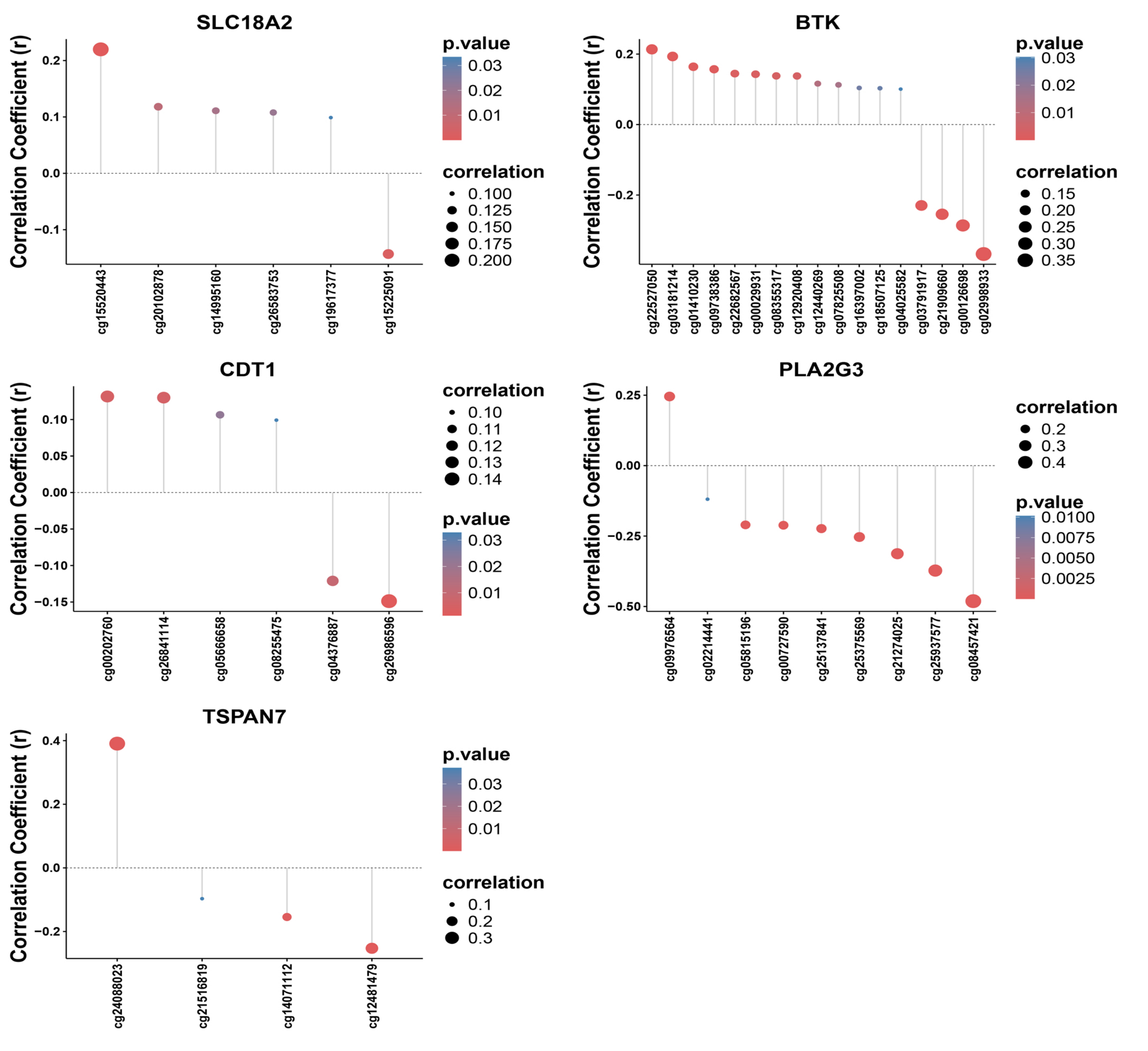

SLC18A2
Correlation Coefficient (r)
0.2
0.1
0.0
−0.1
cg15520443
cg20102878
cg14995160
cg26583753
cg19617377
cg15225091
p.value
0.03
0.02
0.01
correlation
0.100
0.125
0.150
0.175
0.200
BTK
Correlation Coefficient (r)
0.2
0.0
−0.2
cg22527050
cg03181214
cg01410230
cg09738386
cg22682567
cg00029931
cg08355317
cg12920408
cg12440269
cg07825508
cg16397002
cg18507125
cg04025582
cg03791917
cg21909660
cg00126698
cg02998933
p.value
0.03
0.02
0.01
correlation
0.15
0.20
0.25
0.30
0.35
CDT1
Correlation Coefficient (r)
0.10
0.05
0.00
−0.05
−0.10
−0.15
cg00202760
cg26841114
cg05666658
cg08255475
cg04376887
cg26986596
correlation
0.10
0.11
0.12
0.13
0.14
p.value
0.03
0.02
0.01
PLA2G3
Correlation Coefficient (r)
0.25
0.00
−0.25
−0.50
cg09976564
cg02214441
cg05815196
cg00727590
cg25137841
cg25375569
cg21274025
cg25937577
cg08457421
correlation
0.2
0.3
0.4
p.value
0.0100
0.0075
0.0050
0.0025
TSPAN7
Correlation Coefficient (r)
0.4
0.2
0.0
−0.2
cg24088023
cg21516819
cg14071112
cg12481479
p.value
0.03
0.02
0.01
correlation
0.1
0.2
0.3
