# Supplementary material for: Prognostic and immunotherapeutic implications of histamine-related biomarkers in lung adenocarcinoma
Source: PeerJ. 2026 Sep 30;14:e21760. doi: 10.7717/peerj.21760 (PMC13633689; doi:10.7717/peerj.21760)
Supplement: Supplementary file 3 [file peerj-14-21760-s006.pdf]

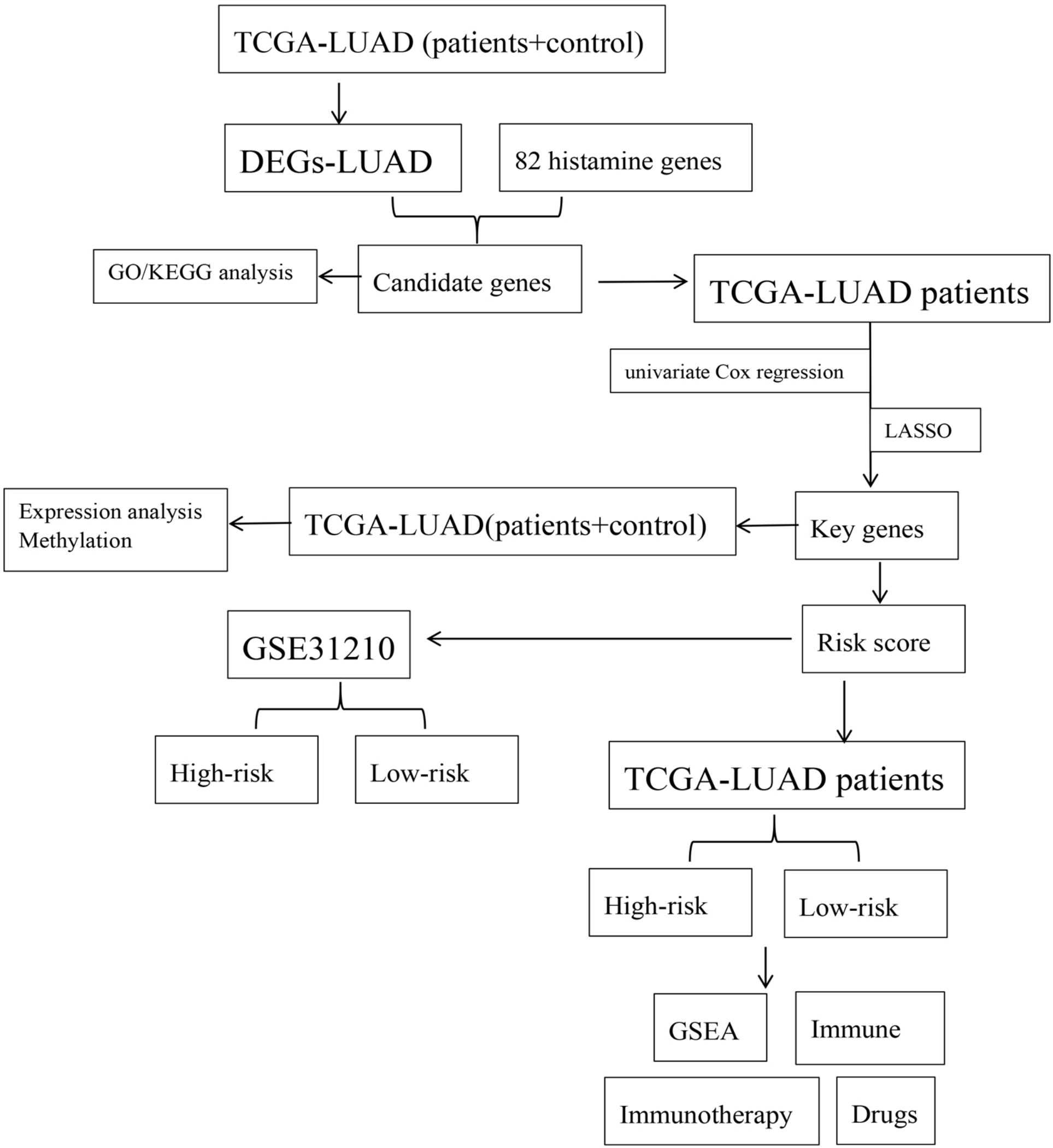
TCGA-LUAD (patients+control)
DEGs-LUAD
82 histamine genes
GO/KEGG analysis
Candidate genes
TCGA-LUAD patients
univariate Cox regression
LASSO
Expression analysis
Methylation
TCGA-LUAD(patients+control)
Key genes
GSE31210
Risk score
High-risk
Low-risk
TCGA-LUAD patients
High-risk
Low-risk
GSEA
Immune
Immunotherapy
Drugs
